# Using the Indirect Cohort Design to Estimate the Effectiveness of the Seven Valent Pneumococcal Conjugate Vaccine in England and Wales

**DOI:** 10.1371/journal.pone.0028435

**Published:** 2011-12-02

**Authors:** Nick Andrews, Pauline A. Waight, Ray Borrow, Shamez Ladhani, Robert C. George, Mary P. E. Slack, Elizabeth Miller

**Affiliations:** 1 Statistics, Modelling and Economics Department, Health Protection Services, Health Protection Agency, London, United Kingdom; 2 Immunisation, Hepatitis and Blood Safety Department, Health Protection Services, Health Protection Agency, London, United Kingdom; 3 Vaccine Evaluation Unit, Microbiology Services Division, Health Protection Agency, Manchester, United Kingdom; 4 Respiratory and Systemic Infection Laboratory, Microbiology Services Division, Health Protection Agency, London, United Kingdom; Universidad Complutense, Spain

## Abstract

**Background:**

The 7-valent pneumococcal conjugate vaccine (PCV-7) was introduced in the United Kingdom in 2006 with a 2,3 and 13month schedule, and has led to large decreases in invasive pneumococcal disease (IPD) caused by the vaccine serotypes in both vaccinated and unvaccinated cohorts. We estimated the effectiveness of PCV-7 against IPD.

**Methods and Findings:**

We used enhanced surveillance data, collated at the Health Protection Agency, on vaccine type (n = 153) and non vaccine type (n = 919) IPD cases eligible for PCV-7. The indirect cohort method, a case-control type design which uses non vaccine type cases as controls, was used to estimate effectiveness of various numbers of doses as well as for each vaccine serotype. Possible bias with this design, caused by differential serotype replacement in vaccinated and unvaccinated individuals, was estimated after deriving formulae to quantify the bias. The results showed good effectiveness, increasing from 56% (95% confidence interval (CI): -7-82) for a single dose given under one year of age to 93% (95% CI: 70-98) for two doses under one year of age plus a booster dose in the second year of life. Serotype specific estimates indicated higher effectiveness against serotypes 4, 14 and 18C and lower effectiveness against 6B. Under the assumption of complete serotype replacement by non vaccine serotypes in carriage, we estimated that effectiveness estimates may be overestimated by about 2 to 5%.

**Conclusions:**

This study shows high effectiveness of PCV-7 under the reduced schedule used in the UK. This finding agrees with the large reductions seen in vaccine type IPD in recent years in England and Wales. The formulae derived to assess the bias of the indirect cohort method for PCV-7 can also be used when using the design for other vaccines that affect carriage such as the recently introduced 13 valent pneumococcal conjugate vaccine.

## Introduction

The 7-valent pneumococcal conjugate vaccine (PCV-7; Prevenar, Pfizer) was first licensed in the United States in 2000 as a 3+1 dose schedule following evidence of high efficacy in children from randomized controlled trials [1]. The vaccine has since been introduced in many countries, including the United Kingdom in September 2006, where a reduced 2 +1 schedule at 2, 4 and13 months was used together with a single dose catch-up for children aged between 12 and 24 months. This reduced schedule was based on immunogenicity rather than efficacy data, and therefore required post-licensure assessment of effectiveness [2].

Within a few years of introduction there was a considerable reduction of vaccine-type invasive pneumococcal disease (VT - IPD) in both the vaccine-targeted age groups and older age groups through herd immunity in England and Wales [3]. Some of these reductions have, however, been offset by increases in non-vaccine type IPD (NVT – IPD) through replacement as documented in a recent review [4]. A higher valency pneumococcal conjugate vaccine (PCV-13, Prevenar-13, Pfizer), was introduced in the UK from April 2010 and contains some of the serotypes that have shown evidence of replacement [5].

The large impact of PCV-7 on IPD suggests the vaccine is highly effective when used outside of trial settings, and this has been confirmed in a number of effectiveness studies evaluating the 3+1 as well as other reduced schedules. A large case-control study in the US and two smaller case-control studies in Spain and Canada have been conducted [6], [7], [8]. In addition, two studies from the US and Germany using the indirect cohort method (also known as the Broome method) have been published [9], [10]. In this paper we assess vaccine effectiveness (VE) of the 2+1 schedule (and alternative reduced dose regimens) against VT-IPD as well as against individual serotypes using enhanced surveillance data from England and Wales. We use the indirect cohort method in which NVT cases serve as controls [11]. This methodology, whilst useful in providing well matched controls, is potentially subject to bias caused by the vaccine increasing the chance of NVT carriage in vaccinated compared to unvaccinated individuals through reduction in VT carriage and replacement by NVT carriage. The extent to which this bias may affect VE estimates has not been considered in previous indirect cohort studies. We derive a formula to estimate the size of this bias and its likely impact on our estimates.

## Methods

### Ethics Statement

The Health Protection Agency has approval under PIAG Section 60 of the Health and Social Act 2001 (which has subsumed into the National Information Governance Board for Health and Social Care with Section 60 –now Section 251 if the NHS Act 2006) to process confidential information from patients for the purposes of monitoring the efficacy and safety of vaccination programmes.

### Study population

We used a database, set up in 1996, in which electronic data on isolates of IPD sent to the Health Protection Agency Respiratory and Systemic Infection Laboratory for serotyping were reconciled with electronic reports of IPD sent to the Health Protection Agency from laboratories in England and Wales [3], [12]. This dataset comprises cases in which *S. pneumoniae* has been identified by culture, or more rarely antigen detection or polymerase chain reaction (PCR), in either cerebrospinal or pleural fluid. Consistent with clinical practice in the UK, blood cultures and cerebrospinal fluid samples are almost exclusively performed on hospitalised patients. From September 2006, vaccination status and clinical information was sought from General Practitioners and Paediatricians treating cases in the dataset that were eligible for vaccination (i.e. individuals born since September 4^th^ 2004). A confirmed case of IPD was defined as a culture of pneumococcus from a normally sterile site, or detection of pneumococcal DNA in pleural or cerebrospinal fluid by dual target (*ply* and *lytA*) polymerase chain reaction (PCR) together with a polysaccharide antigen assay that detects 14 serotypes, including all those in PCV-7 and PCV-13 [13].

In July 2010 we identified all vaccine-eligible cases with a known serotype, a date of IPD from November 2006 to May 2010 and aged ≥5 months. We excluded cases where the episode was known to be a second episode of IPD, where vaccination status was not known and where doses were recorded as being given prior to PCV-7 introduction in September 2006 (for example, in another country). We also excluded cases aged less than 14 months who were part of the one dose catch-up cohort as very few individuals had been vaccinated, and had time for protection to start, by this age.

### Exposure to vaccine

When the vaccine was introduced in September 2006, children who were aged <2 months received the routine 2, 4 and13 month schedule. Children aged 3–8 months were eligible to receive two doses in the period before they were 12 months old followed by the 13 month booster dose. Children aged 8 months to 23 months were eligible for the single dose catch-up once aged over twelve months. Therefore there were a number of different schedules, including partial vaccination whilst under the age of 12 months to evaluate. Table 1 shows the birth cohorts and ages of cases used to evaluate various schedules in the analysis.

**Table 1:** Vaccine schedules according to birth cohort and age of IPD cases within which the schedule was evaluated.

Schedule	Birth cohort	Age of cases for VE
2 dose routine (2,4 months)	July 2006 – December 2009	5 months to <14 months^a^
2 dose older infants (3-8 months)	February 2006 – June 2006	5 months to <14 months
2 dose routine + booster	July 2006 – December 2009	≥14 months
2 dose older infants + booster	February 2006 – June 2006	≥14 months
1 dose catch-up	September 2004-January 2006	≥14 months

a The primary schedule is evaluated using cases up to 14 months of age, after which most children have received a booster dose. The booster and 1 dose catch-up is evaluated with cases ≥14 months as it is scheduled from 13 months.

Vaccine protection was defined as starting 14 days post-vaccination accept for the booster dose where protection was assumed after 7 days.

### Explanatory variables

Information available on each case used in the analysis was cohort (as defined in Table 1), pneumococcal serotype, prematurity (gestation<37 weeks: yes/no), being in a pneumococcal risk group (immunosupressed or asplenic / other risk group/ no risk group), gender, ethnicity (White, Asian, Black African / Caribbean, other) age at illness (5–6months, 7–9months, 10–13months, 14–18months, 19–23months, 24–35months, ≥36months), period of case (November 06-April 07, May 07-October 07, November 07-April 08, May 08-October 08,November 08-April 09, May 09-October 09, November 09-May10) and the main variable of interest, PCV-7 vaccination status. The pneumococcal risk groups were as defined by the UK Department of Health [14].

### Statistical methods

VE was estimated as 1- odds of vaccination in a VT case / odds of vaccination in a NVT case. Multivariable logistic regression was used to adjust for age, gender, period, prematurity and being in a risk group. To investigate whether VE varied by risk group and by prematurity the significance of the interaction between vaccine and these factors was determined. Prematurity and being in a risk group were only included in final models if they were both significant and modified the vaccine effect by more than 5% since data were incomplete for these variables in some subjects. Results are given stratified by cohort and age, and are presented along with 95% confidence intervals (CI).

Serotype-specific VE was also estimated using the indirect cohort method by comparing the odds of vaccination of each vaccine type to the non-vaccine types. To improve precision for this analysis, VE following at least one dose was calculated, which combined data across all cohorts. To test whether VE differed by serotype a Fisher's exact test was used to compare vaccination status within vaccine serotypes. Data analysis was performed in STATA version 10.1.

### Assessment of bias in the indirect cohort method

When using the indirect cohort method it can be shown (Appendix S1) that:
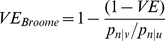
(1)where 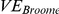 is the observed VE by the indirect cohort method, *VE* is the true vaccine effectiveness, 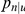 is the probability of non-vaccine type carriage in an unvaccinated individual and 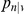 is the probability of non-vaccine type carriage in a vaccinated individual.

Note that in these calculations carriage is probably best interpreted as carriage acquisition rather than carriage prevalence because IPD is thought to occur shortly after acquisition [15]. The two are the same if duration of carriage is the same for vaccine and non vaccine types.

If the probability of non-vaccine type carriage is the same in vaccinated and unvaccinated individuals (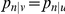) then there is no bias, however it has been shown that the vaccine protects against VT carriage (carriage acquisition) [16], [17] and carriage surveys in the years following vaccination have shown overall carriage prevalence has remained constant with NVT carriage replacing VT carriage [18]. In highly vaccinated populations this replacement occurs in both vaccinated and non-vaccinated individuals through herd immunity. If we assume that carriage replacement starts in vaccinated individuals (who are protected against VT carriage) and then passes to unvaccinated individuals as herd immunity effects occur, then at any point in time 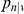 will be greater than 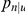 and the indirect cohort method will be biased. If we assume complete replacement at any point in time (which is equivalent to assuming overall carriage rates remain stable and equal in vaccinated and unvaccinated individuals) then it can be shown (Appendix S1) that equation (1) becomes:
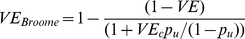
(2)where 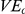 is the effectiveness against carriage and 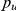 is the proportion of carriage that is vaccine type in the unvaccinated. This formula is used along with estimates for 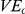 and 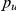 to assess the possible bias in our estimates of VE.

## Results

### Description of IPD cases

**Figure 1:**
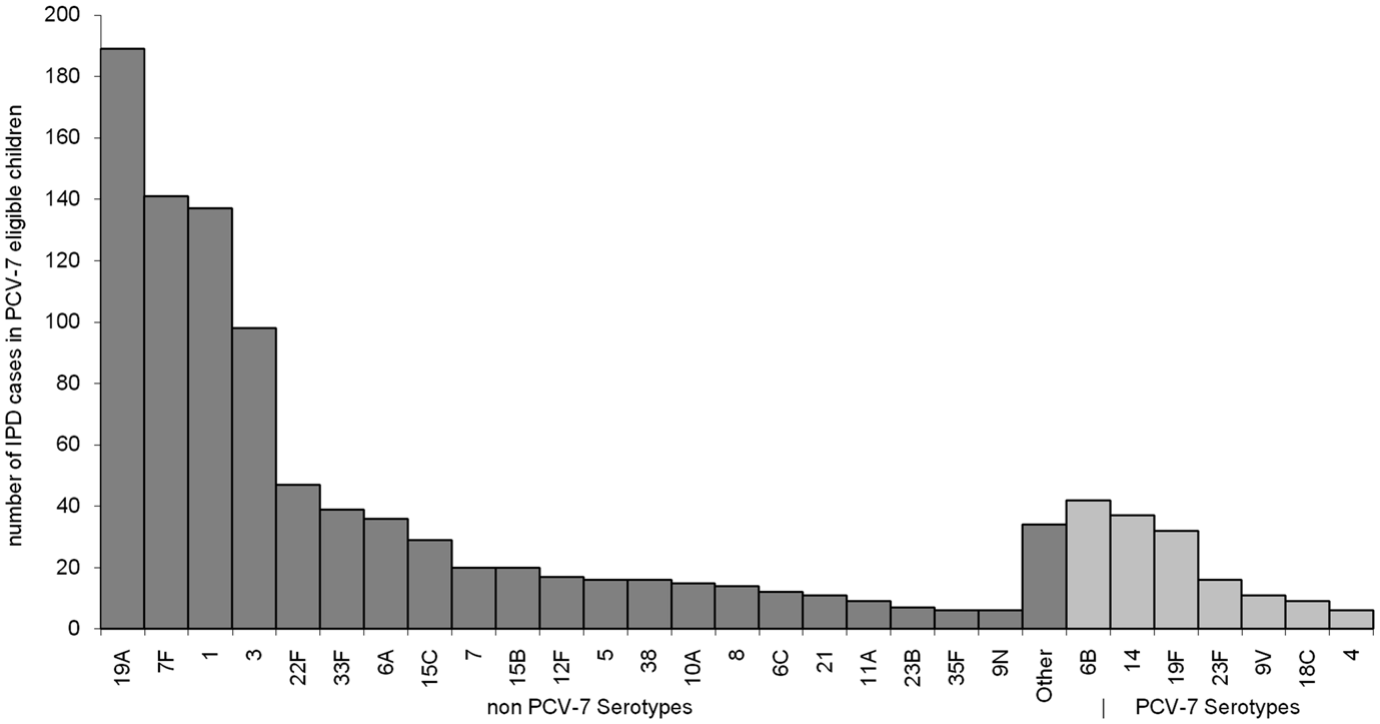
Serotype distribution of IPD cases included in the study. Enhanced surveillance serotyped IPD cases from England and Wales with known vaccination status from November 2006 to May 2010. Dark grey bars are PCV-7 serotypes and light grey bars non PCV-7 serotypes.

Out of a total of 1228 cases in vaccine eligible children aged over 5 months and with onset from November 1^st^ 2006 to May 31^st^ 2010, 1 was dropped as it was a second episode, 101 dropped because they were not serotyped and 13 dropped due to vaccination status unknown or doses given prior to September 2006. Finally 41 were dropped who were part of the one dose-catch-up cohort but aged less than 14 months (none had received the vaccine more than 14 days before onset). This left a total of 1072 IPD cases of which 153 were VT and 919 NVT. Of these cases 127 were diagnosed by PCR only and 945 by culture. The distribution of serotypes is shown in Figure 1, demonstrating a predominance of NVT cases in this post PCV-7 period, with type 19A being the most common. A description of the 153 VT and 919 NVT cases is shown in Table 2. Between November 2006 and May 2010 the number of VT cases fell and the number of NVT cases increased. Vaccine coverage increased over this period and period is, therefore, an important confounding variable. Risk factors were present in a significantly (p = 0.01) greater proportion of the VT cases (23%) than NVT cases (14%). Of the VT cases where prematurity was known 18% were premature compared to 13% of the NVT cases. If we assume those cases where prematurity was unknown were not premature (which is likely because prematurity is likely to be recorded if present) these proportions reduce to 10% in both groups, closer to the national rate of 8.6% [19].

**Table 2:** Description of the 153 VT and 919 NVT IPD cases.

Factor	Level	VT (%)	NVT(%)
Sex	Female	68 (44.4%)	401(43.6%)
	Male	85 (55.6%)	512 (55.7%)
	Missing	0 (0%)	6 (0.7%)
Age	5–6 m	13(8.5%)	38 (4.1%)
	7–9 m	23 (15.0%)	175 (19.0%)
	10–13 m	19 (12.4%)	149 (16.2%)
	14–18 m	31 (20.3%)	141 (15.3%)
	19–23 m	20 (13.1%)	89 (9.7%)
	24–35 m	25 (16.3%)	145 (15.8%)
	> = 36 m	22 (14.4%)	182 (19.8%)
Period	Nov06-April07	67(43.8%)	65 (7.1%)
	May07-Oct07	16 (10.5%)	58 (6.3%)
	Nov07-April08	28 (18.3%)	123 (13.4%)
	May08-Oct08	11 (7.2%)	81 (8.8%)
	Nov08-April09	12 (7.8%)	216 (23.5%)
	May09-Oct09	6 (3.9%)	113 (12.3%)
	Nov09-May10	13 (8.5%)	263 (28.6%)
Ethnicity	Asian	8 (5.2%)	48 (5.2%)
	African/Caribbean	8 (5.2%)	53 (5.8%)
	Other	8 (5.2%)	64 (7.0%)
	White	65 (42.5%)	577 (62.8%)
	Missing	64 (41.2%)	177 (19.3%)
Risk Factor	No	118 (77.1%)	762 (82.9%)
	Immunosupressed / asplenic	15 (9.8%)	53 (5.8%)
	Other risk factor	20 (13.1%)	76 (8.3%)
	Missing	0 (0.0%)	28 (3.0%)
Born Premature	No	74 (48.4%)	631 (68.7%)
	33-36 weeks	12 (7.8%)	57 (6.2%)
	<33 weeks	4 (2.6%)	35 (3.8%)
	Missing	63 (41.2%)	196 (21.3%)

### Vaccine effectiveness

The full break down of vaccination status according to age and doses scheduled is given in Table 3. Numbers were too small for some scenarios to estimate VE so these were either not considered or combined with others. Table 4 shows the VE estimates for various schedules, including partial vaccination. VE is about 80% for two routine doses, 55% for a single dose administered under 12 months of age and 80% for a single dose over 12 months of age in the catch-up campaign. VE for two doses and a booster can be calculated using different eligible vaccinated cohorts which give alternative results. For those scheduled for two routine doses at 2 and 4 months and a booster, the numbers are small and the VE is 62% with a wide 95% CI. However if infants scheduled for primary vaccination at older ages are added VE increases to 93% with a relatively narrow 95% CI. This increased precision is due to the fact that VT IPD incidence was higher in 2006/07 when the doses were scheduled to be given to older infants and also the fact that VE has increased to nearer 100% (which in itself increases precision) through the addition of 7 unvaccinated cases.

**Table 3:** PCV-7 vaccination status of IPD cases by age, scheduled doses and serotype.

Age	Scheduled doses	Vaccination status	VT	NVT
<14 months	2 dose routine	Unvaccinated	5	17
		One dose <12 m	10	47
		Two doses <12 m	18	265
		Total	33	329
	2 dose older infants	Unvaccinated	13	11
		One dose	7	12
		Two doses	2	10
		Total	22	33
≥14 months	2 dose routine + booster	Unvaccinated	1	10
		One dose <12 m	4	7
		Two doses <12 m	3	56
		One dose <12 m + One dose >12 m	0	9
		Fully vaccinated^a^	7	199
		One dose >12 m^b^	0	2
		Total	15	283
	2 dose older infants + booster	Unvaccinated	7	7
		One dose <12 m	2	4
		Two doses <12 m	0	11
		One dose <12 m+ One dose >12 m	0	4
		Fully vaccinated^a^	0	44
		One dose >12 m^b^	0	9
		Total	9	79
	1 dose catch-up	Unvaccinated	53	49
		One dose <12 m	0	2
		Two doses <12 m	0	1
		One dose <12 m+ One dose >12 m	0	2
		Fully vaccinated^a^	0	0
		One dose >12 m^b^	21	141
		Total	74	195

a Fully vaccinated means at least 3 doses with one of them given at an age over 12 months. All but 4 of these are for 2 doses under 12 months and 1 dose over 12 months.

b A total of 4 individuals had more than 2 doses aged >1.

**Table 4:** PCV-7 vaccine effectiveness estimates.

Age of IPD cases	Doses for VE estimate	VT casesvaccinated / total^a^ (%)	NVT casesvaccinated / total^a^ (%)	Crude VE(95% CI)	Adjusted b VE (95% CI)
<14 months	1 dose routine/older infants	17/35 (49%)^c^	59/87 (68%)	55% (1–80)	56% (-7–82)
	2 dose routine	18/23 (78%)	265/282 (94%)	77% (30–92)	79% (24–94)
	2 dose routine/older infants	20/38 (53%)	275/303 (91%)	89% (76–95)	83% (60–93)
≥14 months	2 dose routine + booster	7/8 (88%)	199/209 (95%)	65% (-214–96)	62% (-315–96)
	2 dose routine/older infants + booster	7/15 (47%)	243/260 (93%)	94% (81–98)	93% (70–98)
	1 dose catch-up	21/74 (28%)	141/190 (74%)	86% (75–92)	78% (56–89)

a This is the total either unvaccinated or with the stated schedule.

b Adjusted for age, gender and period.

c 17/35 comes from Table 3 as (10+7)/(10+7+5+13) where 10 and 7 are the one partial dose for routine/older infants and 5 and 13 are the unvaccinated for these groups.

Risk group and prematurity were not included when obtaining adjusted VE estimates as they were not confounding variables, furthermore there was no evidence of interactions between these variables and vaccination status. Although there was no evidence VE differed by prematurity or risk group VE was still evaluated, where numbers were sufficient, within these groups. For prematurity the VE of two doses given under 12 months of age was 93% (95% CI: 72%–98%). For risk groups (combining all together) the VE estimate was 73% (95% CI: 10%–92%) for the one dose catch-up and 91% (95% CI: 36%–99%) for two doses under 12 months of age. For those in the immunosuppressed / asplenic risk group VE was 83% (95%CI: 18%–97%) for the one dose catch-up.

### Serotype specific VE estimates

Serotype specific estimates for at least one dose of vaccine are shown in Table 5. Vaccine effectiveness differed according to serotype (p<0.001) with the highest VE for serotypes 4, 14 and 18C and lowest VE for serotype 6B.

**Table 5:** Serotype specific PCV-7 vaccine effectiveness estimates for at least one dose of vaccine at any age.

Serotype	VT casesvaccinated / total (%)	NVT cases vaccinated / total (%)	Crude VE(95% CI)	Adjusted VE a(95% CI)
4	1/6 (17%)	825/919 (90%)	98% (79–100)	99% (72 to 100)
6B	29/42 (69%)	825/919 (90%)	75% (45–88)	49% (-14–77)
9V	6/11 (55%)	825/919 (90%)	86% (42–97)	79% (-2–96)
14	6/37 (16%)	825/919 (90%)	98% (94–100)	93% (80–98)
18C	3/9 (33%)	825/919 (90%)	94% (73–99)	94% (64–99)
19F	20/32 (63%)	825/919 (90%)	81% (56–91)	70% (29–87)
23F	9/16 (56%)	825/919 (90%)	85% (52–95)	76% (20–93)
All VT	74/153 (48%)	825/919 (90%)	89% (84–93)	79% (67–87)

a Adjusted for age, gender, period and cohort.

### Assessment of bias due to replacement for the indirect cohort method

**Figure 2:**
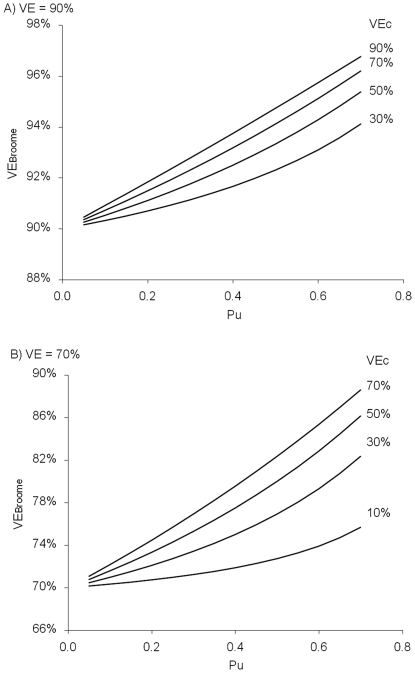
Assessment of bias when estimating VE with the indirect cohort design when there is complete serotype replacement. VE_Broome_ is observed vaccine effectiveness by the indirect cohort method, VE_c_ is true vaccine effectiveness against carriage, VE is true vaccine effectiveness (combining effectiveness against carriage and IPD given carriage) and P_u_ is the proportion of carriage that is VT in the unvaccinated. The formula relating these quantities is VE_Broome_  =  1 – (1-VE)/ (1 + VE_c_ P_u_ / (1-P_u_)). Panel A) VE = 90%, panel B) VE = 70%.

Figure 2 shows the bias when using the indirect cohort method under the assumption of complete replacement for a true VE of 70% and 90% and for various values of VE against carriage (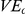) and proportion of carriage that is VT in the unvaccinated (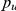). Bias increases as 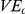 increases and with increasing 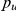. Estimates of effectiveness against acquisition of carriage suggest this is about 50% after a booster dose or two doses given to one year olds [16], [17]. Data on carriage acquisition in unvaccinated individuals over the study period are not available. However data are available on carriage prevalence and show the proportion that were VT fell from 48% to 0% in 5–20 year olds between 2001/02 and 2008/09 [18]. In under 5 year olds in 2001/02 66% of carriage isolates were VT, but no data were available in unvaccinated under 5 year olds in 2008/09 [18]. The proportion of IPD cases in our population that were VT in the unvaccinated fell from about 70% to 10% over the study period, but this may not approximate carriage because case: carrier ratios are higher for VTs [18]. Overall these data would suggest that averaging over the period of this study 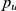 is about 40% ((70%+10%)/2), which along with 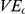  =  50% would mean a true VE of 90% against IPD would be estimated at about 92.5% by the indirect cohort method. A true VE of 70% would be estimated at about 77.5%, however if true VE is only 70% a more realistic value for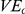 is probably 30% which would yield an observed VE of 75%. Therefore a realistic range for the bias is probably 2 to 5% for this study.

## Discussion

This analysis has shown that PCV-7 has good effectiveness against IPD when used in a reduced schedule in England and Wales. VE of a single dose given under one year of age is about 50%, two doses about 80%, two doses and a booster about 90% and a single dose over one year of age about 75%. These estimates are slightly lower than those reported from the U.S. by Whitney *et al* for similar schedules of 73% (95% CI: 43%–87%) for one dose, 96% (95% CI: 88%–99%) for two doses, 98% (95% CI: 75%–100%) for two doses and a booster and 93% (95% CI: 68%–98%) for 1 dose in toddlers [6]. The U.S. study did, however, report significant effectiveness against NVT IPD which is indicative of residual confounding and hence over estimation of VE. With the exception of a Canadian study where VE following two doses was 99% (95% CI: 90%–100%) [7], our estimates are consistent with those seen in other studies, although these had small numbers and hence wide confidence intervals [8], [9], [10]. The VE estimates are also consistent with the large impact on vaccine type IPD seen in England and Wales, but should be considered in conjuction with the overall impact seen on IPD which is partly offset by serotype replacement [3].

The unadjusted VE estimates obtained by the indirect cohort method were generally similar to the age, gender and period adjusted estimates with the exception of the one dose over one year estimate which reduced from 86% to 78%. This change is due to the reduction in VT cases across a period of increasing vaccine coverage and emphasizes the importance of adjusting for time period when using the indirect cohort method. There were some differences in serotype specific effectiveness, with a particular high VE for serotypes 4, 14 and 18C and lower VE for serotype 6B.

The lower effectiveness for 6B is consistent with the results of studies evaluating the immunogenicity of the two dose priming schedule for PCV7 as used in the UK and of single catch up dose for toddlers [20], [21].

The indirect cohort method has advantages and disadvantages compared to other methods for estimating VE post licensure such as case-control, screening and cohort designs. A cohort design for such a rare disease would require a population based database with IPD serotyping results available. This may be subject to bias if sufficient data on confounding variables are not available. The screening method utilises vaccine coverage data, but requires this to be unbiased and representative of the coverage expected in the population from which the cases arose [22]. This would mean obtaining coverage according to risk factors, exact age and numbers of doses. Case control designs have been employed to estimate PCV-7 VE with controls selected from the same hospitals, health registers and birth registers. The main disadvantage of the case-control method is selection of appropriate controls and the cost of obtaining controls. The advantage of the indirect cohort method over these methods is its efficiency (it only requires serotyped IPD cases) and the fact that the NVT cases should serve as well-matched controls in terms of risk factors and use of health care. One limitation is that the method may underestimate VE if there is cross protection against NVTs. This was evaluated by removal of potential cross-protected serotypes 6A and 19A from the controls and this was found to make very little difference to VE estimates, with the largest change seen for the one dose catch-up were adjusted VE increased from 78% to 81%. The method may also overestimate VE due to protection against VT carriage as evaluated in this paper.

The formula provided in this paper allows calculation of the potential bias when using the indirect cohort study for vaccines that have herd immunity effects through carriage reduction. The bias is at its greatest when the vaccine protection is mainly though carriage reduction, when the vaccine types are still prevalent and when replacement is full (and occurs rapidly). If good data are available on carriage (or carriage acquisition) then VE estimates could be corrected to allow for the bias. In practice such data were not available throughout the study so the bias could only be approximated at about 2–5% under the assumption of an average of 40% of carriage being VT in unvaccinated individuals. This size of bias is small compared to the precision of the VE estimates. In time, as VT carriage reduces through herd immunity the bias reduces, but so do VT cases with which to estimate VE. One consequence of this potential bias would be that vaccinated individuals would have a higher risk of NVT IPD than unvaccinated individuals. A recent US case control study looking at risk factors for IPD reported no such increased risk, although point estimates for the odds ratio were not reported [23].

In summary, our results confirm that PCV-7 is a highly effective vaccine against VT IPD and this has led to large reductions in IPD in children in England and Wales. The indirect cohort method is a useful and efficient design to estimate effectiveness and, for PCV-7 effectiveness estimation, biases associated with the method are likely to be relatively small. The formulae derived to assess the bias of the indirect cohort method can be used for other vaccines that effect carriage such as the recently introduced 13 valent pneumococcal conjugate vaccine.

## Supporting Information

Appendix S1 Derivation of formulae to quantify bias from replacement using the indirect cohort method.
(DOC)
