# Supplementary material for: Using the Indirect Cohort Design to Estimate the Effectiveness of the Seven Valent Pneumococcal Conjugate Vaccine in England and Wales
Source: PLoS One. 2011 Dec 2;6(12):e28435. doi: 10.1371/journal.pone.0028435 (PMC3229580; doi:10.1371/journal.pone.0028435)
Supplement: Appendix S1 — Derivation of formulae to quantify bias from replacement using the indirect cohort method. (DOC) [file pone.0028435.s001.doc]

**Appendix S1**

**Derivation of formulae to quantify bias from replacement using the indirect cohort method**

Define the following:

$VE=$ overall vaccine effectiveness

$VE_{c}=$ vaccine effectiveness against carriage,

$VE_{i}=$ vaccine effectiveness against invasion given carriage,

$p_{v|u}=$probability of vaccine type carriage in an unvaccinated individual (in a unit of time),

$p_{n|u}=$probability of non-vaccine type carriage in an unvaccinated individual,

$p_{v|v}=$probability of vaccine type carriage in a vaccinated individual,

$p_{n|v}=$probability of non-vaccine type carriage in a vaccinated individual,

$p_{iv|u}=$probability of invasion given vaccine type carriage in an unvaccinated individual,

$p_{in|u}=$probability of invasion given non-vaccine type carriage in an unvaccinated individual,

$p_{iv|v}=$probability of invasion given vaccine type carriage in a vaccinated individual,

$p_{in|v}=$probability of invasion given non-vaccine type carriage in a vaccinated individual.

Note that: $VE=1-(1-VE_{c})(1-VE_{i}),$ $p_{v|v}=(1-VE_{c})p_{v|u},$ $p_{iv|v}=(1-VE_{i})p_{iv|u}.$

We assume that the case:carrier ratio of NVT is not affected by vaccination i.e.$p_{in|v}=p_{in|u}$.

Also define the following:

$c=$ vaccine coverage,

$N=$ the denominator population,

$N_{vv}=$the number of vaccine type cases amongst vaccinated individuals (in a unit of time),

$N_{vu}=$ the number of vaccine type cases amongst unvaccinated individuals,

$N_{nv}=$ the number of non-vaccine type cases amongst vaccinated individuals,

$N_{nu}=$the number of non-vaccine type cases amongst unvaccinated individuals.

For the indirect cohort method:

$VE_{Broome}=1-\frac{N_{vv}/N_{vu}}{N_{nv}/N_{nu}}$. (1)

Now:

$$N_{vv}=Ncp_{v|v}p_{iv|v}=Nc(1-VE_{c})p_{v|u}(1-VE_{i})p_{iv|u,}$$

$$N_{nv}=Ncp_{n|v}p_{in|v}=Ncp_{n|v}p_{in|u,}$$

Substituting the above into (1) *N*, *c*, $p_{iv|u}$and$p_{in|u}$ all cancel out, so:

$VE_{Broome}=1-\frac{(1-VE_{c})(1-VE_{i})}{p_{n|v}/p_{n|u}}$ $=1-\frac{(1-VE)}{p_{n|v}/p_{n|u}}$. (2)

So if the probability of non vaccine type carriage in the vaccinated is equal to the unvaccinated there is no bias in the indirect cohort method. But if vaccine reduces carriage then it may be that non vaccine type carriage is higher in the vaccinated than the unvaccinated due to replacement of carriage.

Suppose we assume replacement is complete, which means overall carriage rates do not vary, then:

$$p_{n|v}+p_{v|v}=p_{v|u}+p_{n|u},$$

$$p_{n|v}=p_{v|u}+p_{n|u}-p_{v|v}$$

$$=p_{v|u}+p_{n|u}-(1-VE_{c})p_{v|u}$$

$$=p_{n|u}+VE_{c}p_{v|u}.$$

So: $p_{n|v}/p_{n|u}=1+VE_{c}p_{v|u}/p_{n|u}.$

Substituting in (2) we get:

$VE_{Broome}=1-\frac{(1-VE)}{(1+VE_{c}p_{v|u}/p_{n|u})}$ .

Let$p_{u}=p_{v|u}/(p_{v|u}+p_{n|u})$ = the proportion of carriage that is VT in the unvaccinated then:

$VE_{Broome}=1-\frac{(1-VE)}{(1+VE_{c}p_{u}/(1-p_{u}))}$ .
